# A Muscle Biosignature Differentiating Between Limb-Girdle Muscular Dystrophy and Idiopathic Inflammatory Myopathy on Magnetic Resonance Imaging

**DOI:** 10.3389/fneur.2021.783095

**Published:** 2021-12-20

**Authors:** Wen-Chi Hsu, Yu-Ching Lin, Hai-Hua Chuang, Kun-Yun Yeh, Wing P. Chan, Long-Sun Ro

**Affiliations:** ^1^Department of Medical Imaging and Intervention, Chang Gung Memorial Hospital, Taoyuan, Taiwan; ^2^College of Medicine, Chang Gung University, Taoyuan, Taiwan; ^3^Department of Medical Imaging and Intervention, Chang Gung Memorial Hospital, Keelung, Taiwan; ^4^Department of Family Medicine, Chang Gung Memorial Hospital, Taoyuan, Taiwan; ^5^Division of Hemato-Oncology, Department of Internal Medicine, Chang Gung Memorial Hospital, Keelung, Taiwan; ^6^Department of Radiology, Wan Fang Hospital, Taipei Medical University, Taipei, Taiwan; ^7^Department of Radiology, School of Medicine, College of Medicine, Taipei Medical University, Taipei, Taiwan; ^8^Department of Neurology, Chang Gung Memorial Hospital, Taoyuan, Taiwan

**Keywords:** MRI, muscle atrophy, muscle edema, limb-girdle muscular dystrophy, inflammatory myopathy, fat substitution

## Abstract

**Background:** The overlapping clinical presentations of limb-girdle muscular dystrophy (LGMD) and idiopathic inflammatory myopathy (IIM) make clinical diagnosis challenging. This study provides a comprehensive evaluation of the distributions and characteristics of muscle fat substitution and edema and aims to differentiate those two diseases.

**Methods:** This retrospective study reviewed magnetic resonance imaging (MRI) of seventeen patients with pathologically proved diagnosis, comprising 11 with LGMD and 6 with IIM. The fat-only and water-only images from a Dixon sequence were used to evaluate muscle fat substitution and edema, respectively. The degrees of muscle fat substitution and edema were graded and compared using the appropriate statistical methods.

**Results:** In LGMD, more than 50% of patients had high-grade fat substitution in the majority of muscle groups in the thigh and calf. However, <50% of IIM patients had high-grade fat substitution in all muscle groups. Moreover, LGMD patients had significantly higher grade fat substitution than IIM patients in all large muscle groups (*p* < 0.05). However, there was no significant difference in edema in the majority of muscle groups, except the adductor magnus (*p* = 0.012) and soleus (*p* = 0.009) with higher grade edema in IIM. Additionally, all the adductor magnus muscles in LGMD (100%) showed high-grade fat substitution, but none of them showed high-grade edema.

**Conclusions:** MRI could be a valuable tool to differentiate LGMD from IIM based on the discrepancy in muscle fat substitution, and the adductor magnus muscle could provide a biosignature to categorizing LGMD.

## Introduction

The idiopathic inflammatory myopathy (IIM), such as dermatomyositis and polymyositis, often presents as proximal and symmetric muscle weakness, joint pain, and fatigue (1). The muscle weakness involved the thigh and hip early, then the upper limbs and pharynx, resulting the difficulties climbing stairs, rising from a seated position, or even in swallowing function (2). Its clinical presentations overlap with those of other muscular disorders, such as limb-girdle muscular dystrophy (LGMD). LGMD is an inherited muscular disorder, with the general features of progressive muscle atrophy and weakness (3). The difficulties in climbing stairs or other mobility reduction showed in the patients with LGMD, as those with IIM. In the laboratory test, both diseases show elevated serum creatine kinase (CK) (3–5). Thus, differentiation of LGMD and IIM based on clinical symptoms and signs may be challenging (6–8). Moreover, IIM and LGMD need to be managed in different ways. IIM should be aggressively treated with glucocorticoids and immunosuppressants (9, 10), while LGMD is mainly treated with supportive measures and limb motion preservation (11). Thus, an early diagnosis of IIM or LGMD is essential to prevent delayed treatment.

Although invasive muscle biopsy is the standard diagnostic tool for all muscular disorders, magnetic resonance imaging (MRI) may also be able to play a pivotal role in identifying different muscular disorders. As the primary manifestation of MRI in patients with dermatomyositis or polymyositis, the muscle edema is usually in the proximal and symmetric distribution (12, 13). The hamstrings are usually involved early, followed by the muscles of the buttocks, proximal upper limbs, and neck flexors. The muscle edema is correlated with an active phase of the IIM, but in the chronic stage, fatty infiltration and muscle atrophy could be seen (14–16). In the patients with LGMD, MRI shows fatty infiltration and muscle atrophy as the most salient findings. The involvement of the adductors and semimembranosus muscles could be visualized in several types of LGMD, including 1A, 1B, 2A, 2B, and 2L (17–19). The variance of the degree and distribution of muscle involvement may exist among different types of LGMD (20). Of the common types, the predominant involvement in the posterior thigh muscles demonstrates in LGMD 2A with a sparing of the vastus lateralis muscles until the late stage; but the LGMD 2B shows an early progression to the vastus muscles (17).

Different muscular disorders may show various patterns and degrees of muscle edematous change or muscle atrophy (16). Through the detection of the muscle changes, MRI can identify patients with muscular diseases and reflect the extent of disease involvement (21, 22). However, the literature with direct comparison of the MRI presentations between the IIM and LGMD is scarce, even though the differentiation of the two disease entities based on clinical symptoms and signs could be difficult. Therefore, this study aims to provide a comprehensive evaluation of the distributions and characteristics of muscle fat substitution and edema in both diseases. By understanding the normal muscle patterns and characteristics and the various patterns and extensions of muscle involvement in both diseases, clinicians might be able to make a confirmative diagnosis of LGMD or IIM based on MRI.

## Materials and Methods

### Patients

This study was approved by the Institutional Review Board of Chang Gung Memorial Hospital, with IRB No. 202101135B0 on 12 July 2021 and in compliance with the Health Insurance Portability and Accountability Act. Seventeen patients with muscle biopsy-proven IIM and LGMD between October 2015 and March 2016 were recruited, and MR examinations were reviewed. Four patients had polymyositis, two had dermatomyositis, and 11 had LGMD. Each subtype of LGMD was also recorded if the genetic diagnosis was confirmed. The serum CK level within 6 months before or after the date of MRI in each patient was also collected. Clinical data including sex, age and durations of illness was also reviewed.

### MRI Study

**Figure 1:**
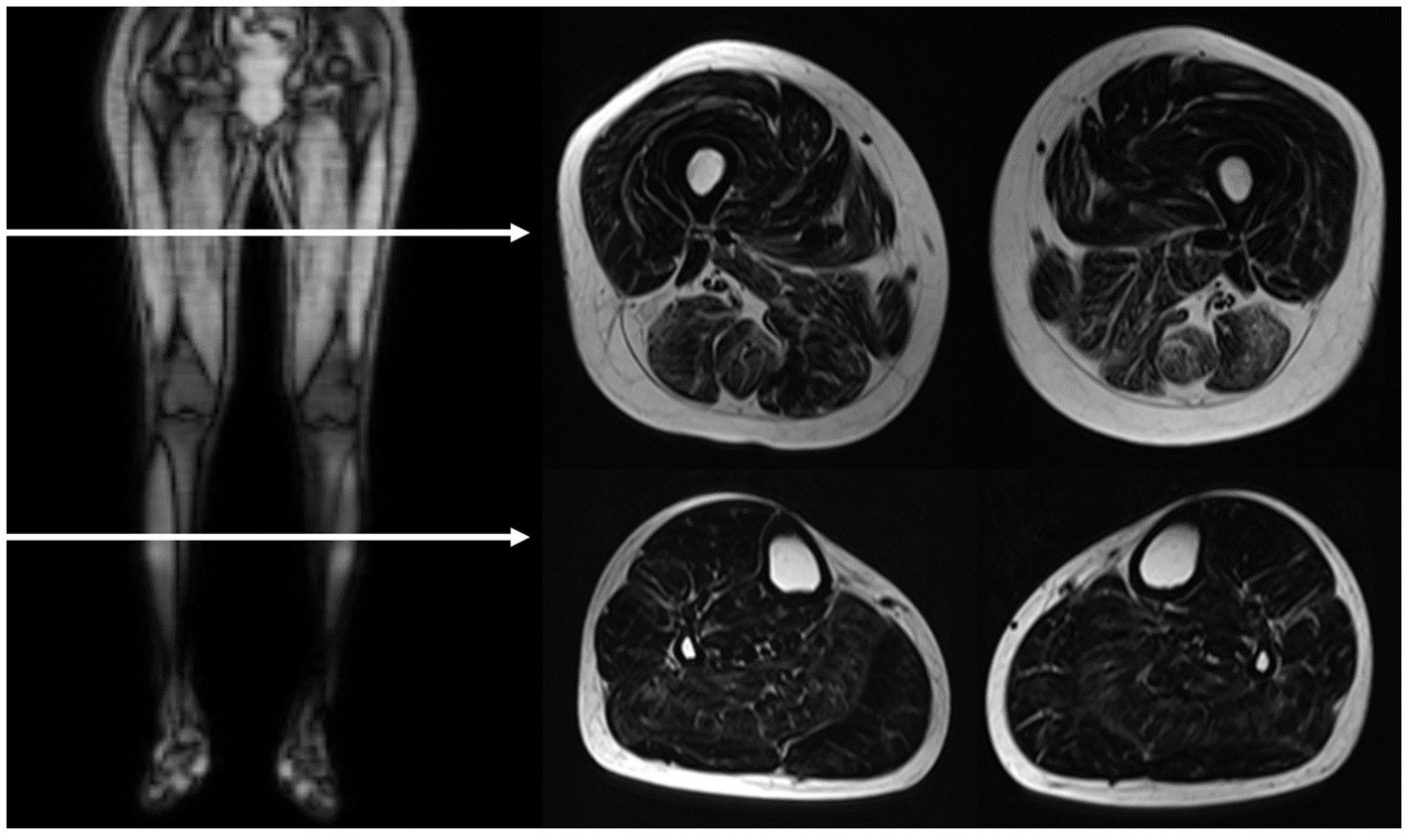
MRI image acquisition. The horizontal arrows in the right side coronal T1-weighted image demonstrated the level of assessed planes, as showed in the left T2-weighted Dixon fat-only axial images. The scanning slices are at the mid-portion of the thigh and the thickest portion of the calf.

MRI of bilateral lower extremities was performed on a 3-tesla scanner (Skyra, SIEMENS, Germany) with surface-coil arrays for signal detection. The standard protocol included a T2-weighted turbo spin-echo Dixon sequence (TR = 4,110 ms, TE = 75 ms, acquisition matrix 256 × 162, slice thickness = 10 mm). Water-only and fat-only images were reconstructed from the Dixon T2 sequences. Each leg was imaged individually. Slices were generally assessed at the mid-portion of the thigh and the thickest portion of the calf (Figure 1).

### Image Analysis

The MRI scans were analyzed by a radiologist with five more years of experience interpreting musculoskeletal imaging and was blind to the clinical information and final diagnosis. The assessment of muscle lesions was based on the distributions and characteristics of muscle involvement and the degrees of muscle fat substitution and edema (13, 19). In the thigh, the following individual muscles in three compartments were identified and analyzed: in the anterior compartment, the vastus lateralis, vastus medialis, rectus femoris, and sartorius; in the medial compartment, the gracilis and adductor magnus; and in the posterior compartment, the semimembranosus, semitendinosus, and biceps femoris. The vastus intermedius is also assessed as a part of the vastus lateralis. The calves included the tibialis anterior, peroneus longus, flexor hallucis longus, lateral and medial gastrocnemius, and soleus. In consideration of the patients' discomfort during the time-consuming MRI containing both T1- and T2-weighted sequences, the T2-weighted Dixon sequence with water-only and fat-only images in the evaluation of muscle edema and fat substitution was applied to shorten the acquisition time. Moreover, there was a high inter-method agreement between the Dixon sequence and T2-weighted short-tau inversion recovery in the evaluation of muscle edema (23). In the grading of muscle involvement (16), the grade of muscle fat substitution was determined by the area of fatty infiltration in Dixon fat-only images. It was categorized into 4 grades as follows: grade 1, no evidence of the presence of fatty infiltration; grade 2, fatty infiltration of <30% of the total muscle area; grade 3, fatty infiltration of between 30 and 60% of the total muscle area; and grade 4, fatty infiltration of more than 60% of the total muscle area (24–26). The degree of individual muscle edema was also determined by the area of involvement assessed by the Dixon water-only images. The grade of edema of each muscle was categorized into 4 grades with the same definitions as fat substitution, but with edema replacing fat substitution in the definitions above. Fat substitution and edematous changes in both legs were recorded, but there was no significant difference between the right and left legs in patients with LGMD and patients with IIM by the Mann-Whitney *U*-test, as shown in Supplementary Tables 1, 2. Therefore, we evaluated only one leg (the left leg) of each patient for further detailed analyses.

**Figure 2:**
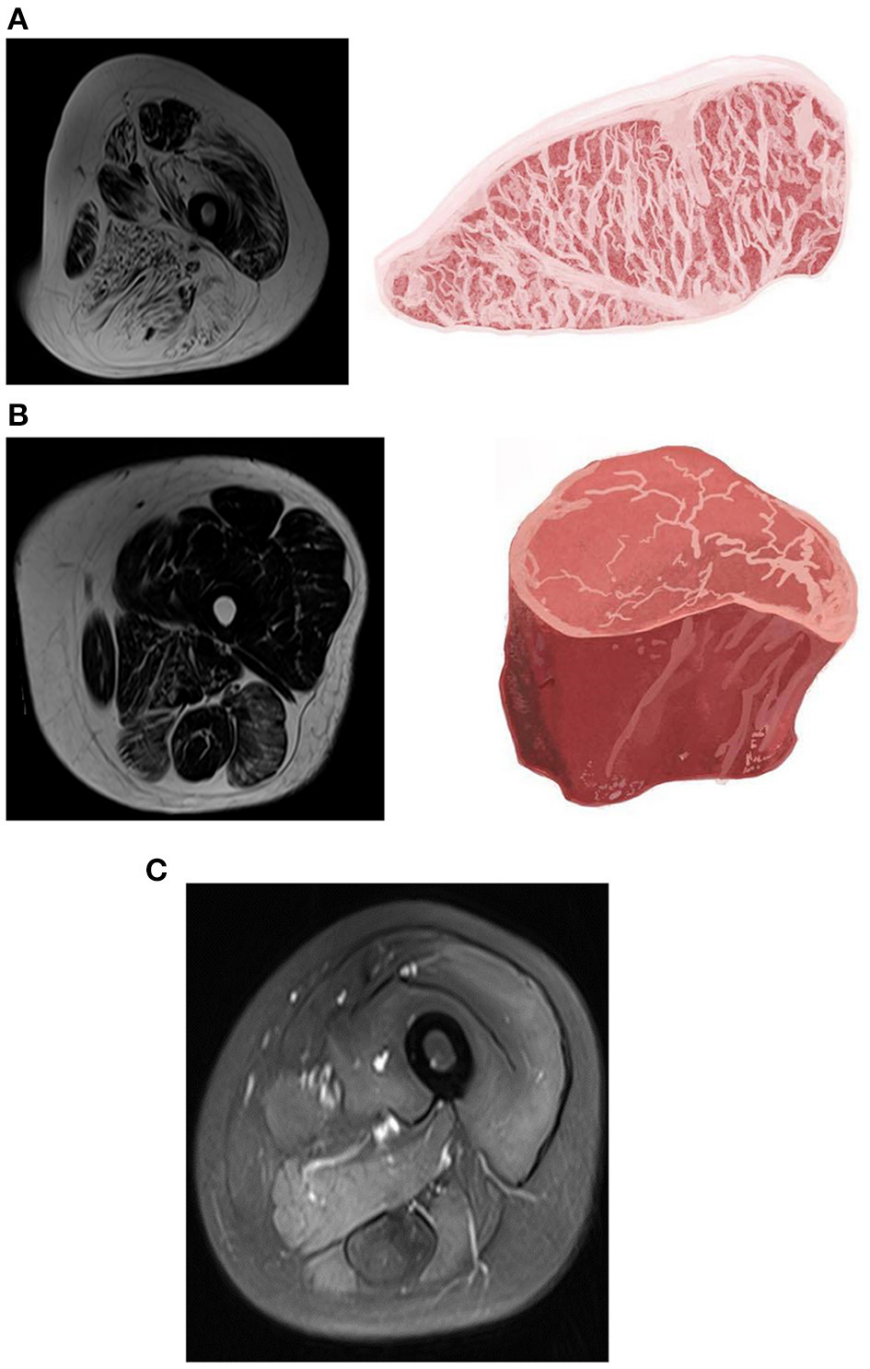
Magnetic resonance imaging (MRI) of muscle fat substitution and edema in different grades and the illustration of Wagyu beef and filet steak. **(A)** T2-weighted Dixon fat-only image of a 40-year-old female with limb-girdle muscular dystrophy (LGMD). There was a high-grade fat substitution in almost all compartments of the thigh muscles, which resembled Japanese A5 Wagyu steak. The marked fatty replacement was noted in the adductor magnus, biceps femoris, semimembranosus, and semitendinosus muscles. **(B)** T2-weighted Dixon fat-only image of a 50-year-old female with polymyositis. Low-grade muscle fat substitution was depicted in IIM compared with LGMD, which resembles the United States Department of Agriculture Choice filet steak. **(C)** T2-weighted Dixon water-only image of a 24-year-old female with polymyositis. High-grade edema in the quadriceps, adductor magnus, semimembranosus, semitendinosus, and biceps femoris.

For a better understanding of the patterns and distributions of the fat substitution and edematous changes in each muscle, grades 3 and 4 of fat substitution were categorized as high-grade fat substitution, featuring abundant intramuscular fatty replacement with marbling distribution in the muscles, reminiscent of Japanese A5 Wagyu beef. In contrast, grades 1 and 2 of muscle fat substitution were categorized as low-grade fat substitution, featuring mild intramuscular fatty replacement resembling United States Department of Agriculture Choice filet steak. Similarly, high-grade and low-grade edema was categorized with an adapted version of the fat substitution grading system above. Figure 2 shows the cases with different grades of muscle fat substitution and edema, as well as the illustration of Wagyu beef and filet steak.

### Statistics

Statistical analyses were performed using the SPSS software package (Version 20.0. Armonk, NY: IBM Corp.). For the description of demographic data, quantitative variables were expressed as the mean ± standard deviation. The differences in the age, duration of illness and CK value between LGMD and IIM were assessed through the two-sample *t*-test. The difference in the sex between the two groups of patients were performed by the Fisher exact test. The differences in fatty infiltration and edema between LGMD and IIM were assessed in terms of median rank through the Mann-Whitney U test. A *p*-value < 0.05 was considered significant.

## Results

A total of 17 patients were included: 11 patients with LGMD and 6 patients with IIM. All the patients with LGMD received the Whole Exome Sequencing (WES) to recognize the subtype of LMGD. There were three patients with LGMD1G, one with 2A, two with 2B, one with 2I, but the rest of four patients with undetermined genotypes. The mean age was 42.6 ± 13.1 (mean ± standard deviation) years in patients with LGMD and 41.8 ± 12.8 years in patients with IIM. There were 5 females (45.45%) in the LGMD group, but the patients with IIM were all females. The mean duration of illness was 9.9 ± 6.2 years in the LGMD group and 2.7 ± 3.2 years in the IIM group. The CK value in the LGMD was 1,859.7 ± 2,089.8 units per liter (U/L), and 571.8 ± 532.6 U/L in the IIM. There was no significant difference in the age (*p*-value = 0.991) and CK value (*p*-value = 0.164) between the LGMD and IIM, but more females in the patients with IIM (*p*-value = 0.043) and longer duration of illness in the patients with LGMD (*p*-value = 0.025) were found.

### Analysis of the Grades of fat Substitution

**Figure 3:**
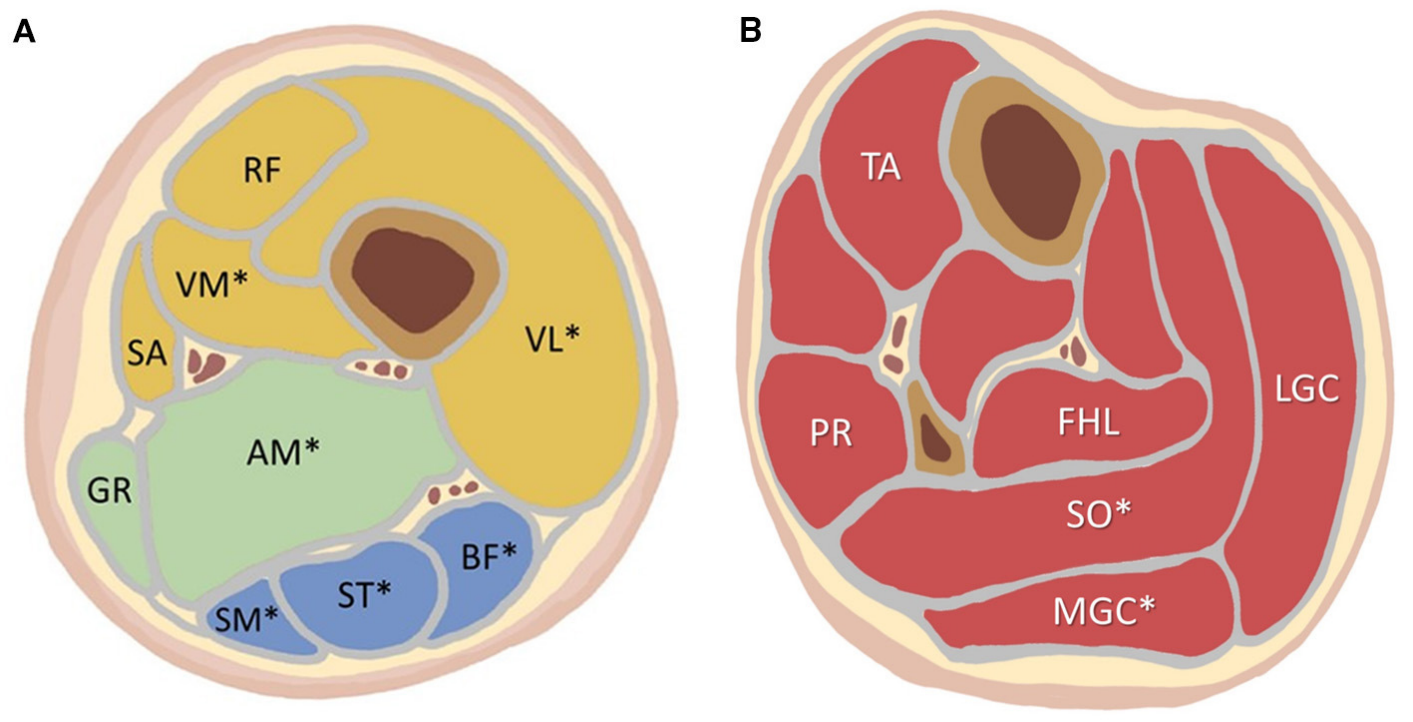
**(A)** The location of each analyzed thigh muscle on the axial imaging. **(B)** The location of each analyzed calf muscle on the axial imaging. More than 50% of the LGMD patients showed high-grade fat substitution in the muscles marked with an asterisk (*): VL, VM, AM, SM, ST, BF, SO, MGC. LGMD, limb-girdle muscular dystrophy; VL, vastus lateralis; VM, vastus medialis; RF, rectus femoris; SA, sartorius; GR, gracilis; AM, adductor magnus; SM, semimembranosus; ST, semitendinosus; BF, biceps femoris; TA, tibialis anterior; PR, peroneus; FHL, flexor hallucis longus; SO, soleus; MGC, medial gastrocnemius; LGC, lateral gastrocnemius.

In the patients with LGMD, no muscle group of the thigh was spared from fat substitution, and high-grade fat substitution was found in the following muscles (Figure 3; Tables 1, 2): in the thigh, the adductor magnus (100%, percentage of patients), vastus medialis (90.0%), biceps femoris (81.8%), semimembranosus (81.8%), semitendinosus (72.7%), and vastus lateralis (72.7%); in the calf, the medial gastrocnemius (63.6%), and soleus (54.5%). Under 50% of patients had high-grade fat substitution in the other muscles of the thigh and calf. For IIM, under 50% of patients had high-grade fat substitution in all muscle groups. Moreover, high-grade fat substitution was found only in the following muscles: in the thigh, the adductor magnus (16.7%), biceps femoris (16.7%), semimembranosus (16.7%), and semitendinosus (16.7%); in the calf, the medial gastrocnemius (16.7%), and soleus (16.7%).

**Table 1:** The percentage of people with high-grade fat substitution and edema of the thigh muscles among patients with limb-girdle muscular dystrophy and idiopathic inflammatory myopathy.

**Analyzed Muscle**	**LGMD**		**IIM**	
	**Fat** **substitution**	**Edema**	**Fat** **substitution**	**Edema**
**Anterior compartment**				
Vastus Lateralis	72.7%	36.4%	0.0%	16.7%
Vastus Medialis	90.9%	27.3%	0.0%	16.7%
Rectus Femoris	45.5%	0.0%	0.0%	16.7%
Sartorius	36.4%	0.0%	0.0%	0.0%
**Medial compartment**				
Gracilis	36.4%	0.0%	0.0%	0.0%
Adductor Magnus	100.0%	0.0%	16.7%	33.3%
**Posterior compartment**				
Semimembranosus	81.8%	36.4%	16.7%	50.0%
Semitendinosus	72.7%	9.1%	16.7%	16.7%
Biceps Femoris	81.8%	18.2%	16.7%	16.7%

*LGMD, limb-girdle muscular dystrophy; IIM, idiopathic inflammatory myopathy*.

**Table 2:** The percentage of people with high-grade fat substitution and edema of the calf muscles among patients with limb-girdle muscular dystrophy and idiopathic inflammatory myopathy.

**Analyzed muscle**	**LGMD**		**IIM**	
	**Fat** **substitution**	**Edema**	**Fat** **substitution**	**Edema**
Tibialis Anterior	36.40%	0.00%	0.00%	0.00%
Peroneus	36.40%	0.00%	0.00%	0.00%
Flexor Hallucis Longus	9.10%	9.10%	0.00%	0.00%
Medial Gastrocnemius	63.60%	18.20%	16.70%	33.30%
Lateral Gastrocnemius	45.50%	18.20%	0.00%	33.30%
Soleus	54.50%	9.10%	16.70%	33.30%

*LGMD, limb-girdle muscular dystrophy; IIM, idiopathic inflammatory myopathy*.

When the grades of fat substitution were compared between LGMD and IIM, most muscle groups in the thighs and calves showed higher grades of fat substitution in the LGMD than IIM (*p*-value < 0.05) as follows: the thigh—the adductor magnus, vastus lateralis, vastus medialis, and all the muscles of the posterior compartment; the calf—the tibialis anterior, medial and lateral gastrocnemius, and soleus (Table 3).

**Table 3:** Comparison of the grades of muscle fat substitution between limb-girdle muscular dystrophy and idiopathic inflammatory myopathy.

**Analyzed muscle**	**Median grade**		***p*** **-value**
	**LGMD**	**IIM**	
**THIGH MUSCLE**			
**Anterior compartment**			
Vastus Lateralis	4	1	0.002^*^
Vastus Medialis	4	1	0.001^*^
Rectus Femoris	1	1	0.155
Sartorius	1	1	0.172
**Medial compartment**			
Gracilis	1	1	0.172
Adductor magnus	4	1.5	<0.001^*^
**Posterior compartment**			
Semimembranosus	4	2	0.016^*^
Semitendinosus	4	2	0.021^*^
Biceps Femoris	4	2	0.003^*^
**CALF MUSCLE**			
Tibialis anterior	2	1	0.033^*^
Peroneus	2	1	0.054
Flexor hallucis longus	1	1	0.173
Medial gastrocnemius	3	1	0.020^*^
Lateral gastrocnemius	2	1	0.004^*^
Soleus	3	1	0.022^*^

* *Significant p-value.*

*LGMD, limb-girdle muscular dystrophy; IIM, idiopathic inflammatory myopathy*.

### Analysis of the Grades of Edema

In LGMD, high-grade muscle edema was found in the following muscles (Figure 3; Tables 1, 2): in the thigh, the semimembranosus (36.4%), vastus lateralis (36.4%), vastus medialis (27.3%), biceps femoris (18.2%), and semitendinosus (9.1%); in the calf, the medial gastrocnemius (18.2%), lateral gastrocnemius (18.2%), flexor hallucis longus (9.1%), and soleus (9.1%). There was a lack of high-grade edema in the other muscles of the thigh and calf. In IIM, high-grade edema was found in the following muscles: in the thigh, the semimembranosus (50.0%), adductor magnus (33.3%), vastus lateralis (16.7%), vastus medialis (16.7%), rectus femoris (16.7%), semitendinosus (16.7%), and biceps femoris (16.7%); in the calf, the medial gastrocnemius (33.3%), lateral gastrocnemius (33.3%), and soleus (33.3%).

When the grades of edema were compared between the LGMD and IIM, most muscle groups in the thighs and calves showed no significant differences, except the adductor magnus (*p*-value = 0.012) and soleus (*p*-value = 0.009), with more severe edema in the patients with IIM than in those with LGMD (Table 4).

**Table 4:** Comparison of the grades of muscle edema between limb-girdle muscular dystrophy and idiopathic inflammatory myopathy.

**Analyzed muscle**	**Median grade**		***p*** **-value**
	**LGMD**	**IIM**	
**THIGH MUSCLE**			
**Anterior compartment**			
Vastus Lateralis	2	1	0.097
Vastus Medialis	1	1	0.345
Rectus Femoris	1	1	0.590
Sartorius	1	1	0.176
**Medial compartment**			
Gracilis	1	1	0.460
Adductor magnus	1	2	0.012^*^
**Posterior compartment**			
Semimembranosus	1	2.5	0.180
Semitendinosus	1	1	1.000
Biceps Femoris	1	1.5	0.650
**CALF MUSCLE**			
Tibialis anterior	1	1	0.460
Peroneus	1	1	0.281
Flexor hallucis longus	1	1	0.173
Medial gastrocnemius	2	2	0.669
Lateral gastrocnemius	1	1	0.906
Soleus	1	2	0.009^*^

* *Significant p-value.*

*LGMD, limb-girdle muscular dystrophy; IIM, idiopathic inflammatory myopathy*.

## Discussion

The overlapping clinical presentations and imaging findings in LGMD and IIM can easily lead to misdiagnosis of these diseases in clinical practice (6–8). The comparison in the age and CK value in our study demonstrated no significant difference, but there were more females in IIM and longer duration of illness in LGMD. This study aims to provide a comprehensive evaluation of the distributions and characteristics of muscle fat substitution and edema in both diseases. LGMD had significantly higher grade fat substitution in the majority of muscle groups of the thigh and calf, except for smaller muscle sizes, such as the rectus femoris, sartorius, gracilis, peroneus, and flexor hallucis longus. However, there were no significant differences in edema among most of the muscle groups between the two disease entities, except the adductor magnus and soleus showed higher grades of muscle edema in IIM. Furthermore, all the adductor magnus muscles showed high-grade fat substitution in the patients with LGMD, but none of the adductor magnus muscles showed high-grade edema. Therefore, the adductor magnus muscle could be considered a biosignature muscle in categorizing LGMD. LGMD should be first considered if there is high-grade fat substitution and almost no edema in the adductor magnus muscle.

In previous reports, the most salient MRI findings for LGMD were muscle wasting and fat replacement, and the IIM was muscle edema (14, 16, 17, 19, 27). Although muscle wasting and fat replacement eventually occur in the chronic stage of IIM (14, 16, 28), the degree of wasting may still be different between LGMD and IIM. In this study, both diseases were in a chronic stage, and fat replacement within the muscle of the LGMD was remarkably abundant, resembling Japanese A5 Wagyu beef (Figure 2). In contrast, the development of severe and diffuse fat substitution was not seen in the chronic stage of IIM. The degree of muscle fatty replacement was significantly lower in the patients with IIM, resembling leaner filet steak. Thus, the degree of muscle fat substitution may consider a biomarker to differentiate LGMD from IIM.

The literature comparing muscle edema between LGMD and IIM is scarce. It was previously reported that muscle edema could be found in IIM, especially in the active stage of disease (14, 15). However, in our study, there was no significant difference in muscle edema of most of the lower limb muscle groups between the LGMD and IIM, except the adductor magnus and soleus muscles. The edematous change indicates the active phase of myositis. The mild degree of muscle edema in both disease entities might be related to the chronic disease status. However, the early stage of LGMD and IIM may be clinically silent, and most of them were diagnosed only in the subacute or chronic stage (29–31). However, further investigations of muscle edema in an acute stage of LGMD and IIM are warranted.

Notably, this study demonstrated that the adductor magnus could be a good biomarker to differentiate LGMD from IIM. Previously, muscle wasting followed a specific pattern in various LGMDs and might help categorize different subtypes of LGMD. For example, in LGMD 2I, the adductor and biceps femoris muscles are commonly involved muscle groups, followed by the rest of the thigh muscles. At the same time, the gracilis and sartorius muscles were relatively spared (19, 32). Moreover, apparent wasting of the adductor muscle with fatty infiltration could be found in numerous LGMD subtypes, such as 1A, 1C, 1D, 2A, 2B, 2C, 2I, and R1 (13, 17–19, 32–34). The above findings are consistent with our study, suggesting that the adductor magnus muscle showed high-grade fat substitution in all patients with LGMD. Thus, the adductor magnus muscle might be the most vulnerable muscle in LGMD that consequently shows high-grade fat substitution but an absence of edema. Moreover, adductor magnus muscle is an easily identified muscle. Therefore, the adductor magnus could be a biosignature muscle in categorizing LGMD with a characteristic of high-grade muscle fat substitution and lack of muscle edema. To better realize the imaging characteristics in the two diseases, the summary was presented in Table 5.

**Table 5:** The brief summary of the imaging characteristics in the patients with limb-girdle muscular dystrophy and idiopathic inflammatory myopathy.

	**LGMD**	**IIM**
Edema	Low grade in most muscles	Low grade in most muscles
Fat substitution	High grade in most muscles	Low grade in most muscles
Biosignature muscle	Adductor magnus	No

*LGMD, limb-girdle muscular dystrophy; IIM, idiopathic inflammatory myopathy*.

However, there are still some limitations in the study. First, a relatively small sample size without an age-matched study design may not be appropriate to generalize the findings to the entire population. To verify the diagnostic accuracy of the imaging biomarker, a prospective, large number, age-matched and blind study is warranted. Second, because of the retrospective nature of this study, the interval from disease onset to MRI studies varied greatly, and most patients had a chronic disease status. Nevertheless, all of our patients were diagnosed with a definitive pathology by muscle biopsy. Third, the semi-quantitative evaluation of edema and fat substitution was used in our study, rather than a quantitative approach with precise estimation of the fat fraction. However, the semi-quantitative method may be more feasible in daily clinical practice without the need for an additional software assistance. Fourth, the bias cannot be avoided with the imaging interpretation form one single radiologist. Nonetheless, previous literature showed the inter-rater agreements were acceptable in the MRI evaluation of fat substitution and muscle edema (35). Fifth, instead of thinner thickness, we acquired 10 mm slice thickness acquisition for all our scans to shorten the scanning time. Lastly, there were three patients with LGMD1G, one with 2A, two with 2B, one with 2I, but the rest of four patients with undetermined genotypes. However, literature reported up to 50% of clinically defined LGMD cases are without a genetic diagnosis, despite intensive research and much effort on the identification of novel causative genes (36). Furthermore, we tried to figure out the imaging characteristics of most of the patients with LGMD rather than the specific features of each subtype, because our study aimed to differentiate between the LGMD and IIM, which have different treatments.

## Conclusions

MRI Dixon imaging was demonstrated to be a useful diagnostic tool in differentiating LGMD from IIM based on the degree of muscle fat substitution in various muscle groups. LGMD patients had significantly higher grade fat substitution than IIM patients in most of the muscle groups of the thighs and calves. However, there was no significant difference in edema among most of the muscle groups between the two diseases. Notably, the adductor magnus appears to be useful as a biosignature muscle in categorizing LGMD. LGMD should be first considered if there is high-grade fat substitution and almost no edema in the adductor magnus muscle.

## Data Availability Statement

The original contributions presented in the study are included in the article/Supplementary Material, further inquiries can be directed to the corresponding author/s.

## Ethics Statement

The studies involving human participants were reviewed and approved by Institutional Review Board of Chang Gung Memorial Hospital with IRB No. 202101135B0 on 12 July 2021. Written informed consent for participation was not required for this study in accordance with the national legislation and the institutional requirements. Written informed consent was not obtained from the individual(s) for the publication of any potentially identifiable images or data included in this article.

## Author Contributions

W-CH and Y-CL: conceptualization, formal analysis, investigation, methodology, and original draft writing. H-HC, K-YY, and WC: resources and draft review and editing. L-SR: conceptualization, supervision, validation, and draft review and editing. All authors contributed to the article and approved the submitted version.

## Conflict of Interest

The authors declare that the research was conducted in the absence of any commercial or financial relationships that could be construed as a potential conflict of interest.

## Publisher's Note

All claims expressed in this article are solely those of the authors and do not necessarily represent those of their affiliated organizations, or those of the publisher, the editors and the reviewers. Any product that may be evaluated in this article, or claim that may be made by its manufacturer, is not guaranteed or endorsed by the publisher.
