# Supplementary material for: A Muscle Biosignature Differentiating Between Limb-Girdle Muscular Dystrophy and Idiopathic Inflammatory Myopathy on Magnetic Resonance Imaging
Source: Front Neurol. 2021 Dec 20;12:783095. doi: 10.3389/fneur.2021.783095 (PMC8720967; doi:10.3389/fneur.2021.783095)
Supplement: Supplementary file 1 [file Table_1.DOCX]

Supplementary Material

# Supplementary Table 1. Comparison of the right and left muscle groups in the patients with limb-girdle muscular dystrophy.

| Muscle Groups | P-value | |
| --- | --- | --- |
|  | Atrophy | Edema |
| Vastus Lateralis | 0.85 | 1.00 |
| Vastus Medialis | 1.00 | 1.00 |
| Rectus Femoris | 1.00 | 0.13 |
| Sartorius | 0.85 | 1.00 |
| Gracilis | 1.00 | 1.00 |
| Adductor magnus | 0.75 | 1.00 |
| Semimembranosus | 0.90 | 0.33 |
| Semitendinosus | 1.00 | 0.48 |
| Biceps Femoris | 0.75 | 0.48 |
| Tibialis anterior | 0.85 | 0.70 |
| Peroneus | 0.85 | 0.95 |
| Flexor hallucis longus | 0.80 | 0.90 |
| Medial gastrocnemius | 0.70 | 1.00 |
| Lateral gastrocnemius | 1.00 | 0.65 |
| Soleus | 0.85 | 0.80 |

# Supplementary Table 2. Comparison of the right and left muscle groups in the patients with idiopathic inflammatory myopathy.

| Muscle Groups | P-value | |
| --- | --- | --- |
|  | Atrophy | Edema |
| Vastus Lateralis | 1.00 | 0.49 |
| Vastus Medialis | 1.00 | 1.00 |
| Rectus Femoris | 1.00 | 1.00 |
| Sartorius | 1.00 | 0.70 |
| Gracilis | 1.00 | 1.00 |
| Adductor magnus | 0.59 | 0.82 |
| Semimembranosus | 0.49 | 0.07 |
| Semitendinosus | 0.49 | 0.70 |
| Biceps Femoris | 0.49 | 0.82 |
| Tibialis anterior | 1.00 | 1.00 |
| Peroneus | 1.00 | 1.00 |
| Flexor hallucis longus | 1.00 | 1.00 |
| Medial gastrocnemius | 0.94 | 0.59 |
| Lateral gastrocnemius | 1.00 | 0.70 |
| Soleus | 0.70 | 0.82 |
